# Leukemic infiltration of the optic nerve in chronic lymphocytic leukemia: A case report and review of literature

**DOI:** 10.1016/j.lrr.2023.100391

**Published:** 2023-08-28

**Authors:** Louisa Liu, Sana Hadyah, Annie Park, Mojtaba Akhtari, Jonathan Scott, Dani Ran-Castillo, Esther Chong, Han Koh, Karlos Oregel, Keerti Khandelwal, Rahel Demisse

**Affiliations:** a Department of Internal Medicine, University of California, Riverside School of Medicine, Riverside, CA, United States of America; b Department of Medical Oncology/Hematology, Loma Linda University Medical Center, Loma Linda, California, United States of America

**Keywords:** Chronic lymphocytic leukemia, Optic neuropathy, Leukemic infiltration, Optic nerve infiltration

## Abstract

Ophthalmic and neurologic involvement are rare complications of CLL, with few cases reported in the literature. We report a case of CLL with leukemic infiltration of the optic nerve and review of literature focusing on management and outcomes. A patient with heavily pretreated CLL presented to our hospital with progressive eye pain and was found to have infiltrative optic neuritis. CSF analysis confirmed involvement with CLL. After systemic treatment with R-CHOP and high-dose methotrexate, along with intrathecal cytarabine and hydrocortisone, she experienced significant improvement and was discharged home. Given the rarity of ophthalmic involvement in CLL, we reviewed all 15 previously reported cases of CLL with optic neuropathy as the first manifestation of CNS involvement and discussed the range of treatment options used and their respective outcomes.

## 1 Introduction

Central nervous system (CNS) involvement is a rare complication of chronic lymphocytic leukemia (CLL) with an incidence of 0.8%−2% and includes manifestations such as cognitive impairment, cerebellar dysfunction, and cranial nerve palsies [1], [2], [3]. Ophthalmic disease in CLL is uncommon with 15 cases of optic nerve infiltration reported [1], [2], [3], [4], [5], [6], [7], [8], [9], [10], [11], [12], [13], [14], [15]. We contribute one additional case of infiltrative optic neuritis in a CLL along with a comprehensive literature review. A systematic search was conducted in the PubMed database to identify all case reports.

## 2 Case presentation

A 49-year-old woman was diagnosed with CLL seven years ago via axillary lymph node (LN) biopsy. Pathology was consistent with CLL/small lymphocytic lymphoma (SLL); immunohistochemical characterization demonstrated CD20(+), PAX5(intranuclear+), CD5(+), CD10(-), CD29(+), CD43(+), BCL1(-), BCL2(+), BCL6(-), MUM1(-). Ki-67 immunostaining demonstrated a low fraction of proliferating cells (<5%). Bone marrow (BM) biopsy revealed 70% involvement by neoplastic B-cells expressing CD5, CD19, CD20, CD22, CD23, CD45, HLA-DR..+, sig kappa+, FISH 13q deletion, and normal cytogenetics. Records of immunoglobulin heavy chain variable gene (IGHV), TP53, CD38, ZAP70, and CD49d were unavailable. She was diagnosed with CLL Rai stage IV and treated with six cycles of Rituximab, Cyclophosphamide, Vincristine, Prednisolone with good clinical response. Two years later, she developed worsening lymphadenopathy with splenomegaly. Repeat LN biopsy was consistent with CLL/SLL. She underwent six cycles of Rituximab and Bendamustine with good response. Three years later, she developed worsening lymphadenopathy. BM biopsy revealed hypercellular marrow with 90% involvement by leukemic cells. She started Ibrutinib, but was discontinued due to severe transaminitis. Acalabrutinib was started, with good clinical response, however was discontinued after seven months per patient preference.

Five months later, she developed progressive bilateral eye pain and blurry vision. Examination revealed diminished visual acuity, optic disk edema, but normal ocular pressures. Optical coherence tomography displayed elevated nerve fiber layer optic nerve thickness in oculus dexter. Brain magnetic resonance imaging (MRI) showed ill-defined enhancement of the right optic nerve. Ophthalmology deferred optic nerve biopsy because of likely visual impairment.

Cerebral spinal fluid (CSF) analysis revealed abnormal CD5+ B*-*cell population (67% of sample), immunophenotype CD45+, CD19+, CD20+, CD10-, CD23+, FMC7-, CD30-, CD38-, CD43+, HLA-DR..+, and sig kappa+. BM biopsy revealed CD5+ monotypic B-cell population (74%) with CLL/SLL. Cytogenetic analysis revealed trisomy 12. Complete blood count showed leukocytosis and absolute lymphocytosis with white blood count (WBC) 15 × 10^3^/µL, normocytic anemia with hemoglobin (Hgb) 11 g/dL, and platelets 168 × 10^3^/µL. Right inguinal LN biopsy revealed atypical lymphoid infiltrate without transformation to a higher-grade lymphoma. She was started on IT-MTX with plans to continue treatment until CSF cleared, with systemic therapy with Rituximab and Venetoclax under consideration.

The patient underwent two cycles of IT-MTX and three cycles of intravitreal MTX. Acute encephalopathy and hypercapnic respiratory failure complicated plans, requiring intubation due to vocal cord edema from suspected CLL infiltration. Computed tomography (CT) head revealed diffuse cerebral edema and possible brainstem herniation. MRI brain demonstrated patchy meningeal enhancement possibly due to carcinomatosis. She developed severe pancytopenia: WBC 5 × 10^3^/µL, Hgb 6.1 g/dL, platelets 26 × 10^3^/µL, and absolute neutrophil count 0/L, likely due to disease burden and MTX. Repeat CSF analysis revealed 60% CD5+/CD10-kappa-restricted monotypic B-cells. She started systemic therapy with R-CHOP alternating with high-dose MTX (HD-MTX) and IT Cytarabine and Hydrocortisone. After two cycles of R-CHOP, three cycles of HD-MTX, and three cycles of IT-Cytarabine, repeat CSF analysis revealed 41.4% CD5+/CD10-kappa restricted B-cells. Her overall performance status and vision significantly improved, and she is undergoing evaluation for BM transplantation.

## 3 Discussion

In our review, we observed a wide range of treatment modalities, reflecting the lack of consensus regarding treatment of CNS involvement in CLL. We identified 15 CLL patients who presented with optic neuropathy as the first manifestation of CNS involvement (Table 1). Treatment is often extrapolated from cases of CLL with other forms of CNS involvement. As such, we included cases of CLL with neurologic disturbances outside of optic neuropathy.

**Table 1:** Summary of All Cases of CLL with Optic Neuropathy as Initial Manifestation of CNS Involvement.




**Abbreviations:** BR: bendamustine and rituximab; CNS: central nervous system; CT: computed tomography; FR: fludarabine and rituximab; Hb: hemoglobin; Hct: hematocrit; HD-MTX: high-dose methotrexate; IT: intrathecal; IV: intravenous; MRI: magnetic resonance imaging; OD: oculus dexter; OS: oculus sinister; PO: by mouth; RAPD: relative afferent pupillary defect; R-CHOP: rituximab, cyclophosphamide, doxorubicin, vincristine, prednisolone; RCV-P: rituximab, cyclophosphamide, vincristine, prednisolone; R-FC: rituximab-fludarabine, cyclophosphamide; TID: three times a day.

Historically, first-line treatment options included optic nerve or whole-brain radiation, which carried prohibitive risks of cognitive side effects in older patients. Recently, IT chemotherapy alone has become a primary treatment for leptomeningeal disease in CLL for its rapid antitumoral effect on CNS dissemination, long duration of action, and longer survival compared with added cranial irradiation [2,5,16]. The most commonly used IT agents are MTX, Cytarabine, and corticosteroids [5,17,18].

Other studies have used systemic chemotherapy that are standard treatments for non-high-risk CLL, including Fludarabine, Cyclophosphamide, HD-MTX, and Vincristine, both with and without IT chemotherapy. Fludarabine-based immunochemotherapy has achieved durable responses in many CLL patients with CNS involvement, including those who failed rituximab monotherapy, intravenous (IV) immunoglobulin, or IV corticosteroids [4]. Given its demonstrated success, the use of chemotherapy in the setting of CNS involvement in CLL has been extrapolated to cases of CLL with optic neuropathy [19]. Newer agents, such as Ibrutinib, Venetoclax, and Lenalidomide, have also shown promising efficacy against CNS lesions given their ability to cross the blood-brain barrier (BBB) and achieve response in cases of B-cell malignancy with CNS involvement [4,[13], [14], [15],[20], [21], [22]].

Our patient was treated for CLL with various therapeutic regimens, lost to follow up several times, and developed optic neuropathy six years after initial CLL diagnosis. We elected to treat her with systemic Venetoclax and Rituximab with intrathecal and intravitreal-MTX given the acuity of symptoms, long treatment course, and demonstrated liver toxicity with Ibrutinib. After a complicated course on IT-MTX, she was instead started on R-CHOP and HD-MTX with IT-Cytarabine and Hydrocortisone.

The addition of HD-MTX to R-CHOP is routinely used as prophylaxis and treatment of DLBCL at risk for CNS relapse and CNS involvement at presentation, respectively. The patient had no biopsy-proven transformation to high-grade lymphoma. Instead, the decision to start R-CHOP and HD-MTX was made to provide systemic anti-tumor activity and effective BBB penetration. CNS involvement may be the only sign of progression of CLL and can be considered a criterion for initiation of systemic chemotherapy, which can control systemic disease and in turn, meningeal involvement [4]. Indeed, several cases have demonstrated success with R-CHOP in CLL with leptomeningeal involvement in the absence of transformation [23,24]. Given the patient's rapidly progressive disease evidenced by cytopenia, she was started on systemic therapy with R-CHOP. However, R-CHOP has its limitations, including poor BBB penetration and inability to achieve sufficient concentrations in the CSF [25]. Consequently, HD-MTX was initiated in combination with R-CHOP. MTX, when given in high doses, can cross the BBB and reach the CNS at an effective concentration to control leptomeningeal metastasis and neurologic symptoms. Intrathecal Cytarabine was chosen due to its efficacy in the treatment of CNS infiltration. Intrathecal Hydrocortisone has been shown to have a synergistic effect with intrathecal Cytarabine and is thought to attenuate arachnoiditis associated with Cytarabine administration.

Improvement in the patient's visual symptoms and CLL clone in her CSF was encouraging. Further follow-up will be needed clinically along with repeat imaging. As confirmed by our literature review, treatment for CNS involvement in CLL has changed considerably in the last few decades. Given the rarity of the phenomenon and limited data available, the best treatment for this patient group remains unclear. As such, the aggregation of existing and future case data is ultimately needed to standardize care and improve prognosis in this disease.

## Author contributions

L.L participated in the care of the patient, conceived the idea for publication, drafted and edited the manuscript; S.H. drafted the manuscript; A.P. participated in the care of the patient and provided edits to the manuscript; M.A., J.S., D.R., E.C., H.K., K.O. provided edits and expertise to the manuscript; K.K. and R.D. participated in the care of the patient, provided edits and expertise to the manuscript.

## Consent

The patient provided informed consent for the inclusion of her data in this report. The patient understood that the results will be fully anonymized, and she cannot be identified via this report.

## Declaration of Competing Interest

The authors declare no competing financial interests.
